# Comparison of ELISA- and SIMOA-based quantification of plasma Aβ ratios for early detection of cerebral amyloidosis

**DOI:** 10.1186/s13195-020-00728-w

**Published:** 2020-12-05

**Authors:** Steffi De Meyer, Jolien M. Schaeverbeke, Inge M. W. Verberk, Benjamin Gille, Maxim De Schaepdryver, Emma S. Luckett, Silvy Gabel, Rose Bruffaerts, Kimberley Mauroo, Elisabeth H. Thijssen, Erik Stoops, Hugo M. Vanderstichele, Charlotte E. Teunissen, Rik Vandenberghe, Koen Poesen

**Affiliations:** 1 Laboratory for Molecular Neurobiomarker Research, Department of Neurosciences, KU Leuven, box 7003, Herestraat 49, 3000 Leuven, Belgium; 2 Laboratory Medicine, UZ Leuven, Leuven, Belgium; 3 Leuven Brain Institute (LBI), KU Leuven, Leuven, Belgium; 4 Laboratory for Cognitive Neurology, Department of Neurosciences, KU Leuven, Leuven, Belgium; 5 Neurochemistry Laboratory, Department of Clinical Chemistry, Amsterdam UMC, Amsterdam, The Netherlands; 6 Neurology Department, UZ Leuven, Leuven, Belgium; 7 ADx NeuroSciences, Ghent, Belgium; 8 Biomarkable, Ghent, Belgium

**Keywords:** Preclinical Alzheimer’s disease, Plasma, β-Amyloid, Biomarkers, Immunoassay, ELISA, SIMOA, Cerebral amyloidosis, Prescreening

## Abstract

**Background:**

Blood-based amyloid biomarkers may provide a non-invasive, cost-effective and scalable manner for detecting cerebral amyloidosis in early disease stages.

**Methods:**

In this prospective cross-sectional study, we quantified plasma Aβ_1–42_/Aβ_1–40_ ratios with both routinely available ELISAs and novel SIMOA Amyblood assays, and provided a head-to-head comparison of their performances to detect cerebral amyloidosis in a nondemented elderly cohort (*n* = 199). Participants were stratified according to amyloid-PET status, and the performance of plasma Aβ_1–42_/Aβ_1–40_ to detect cerebral amyloidosis was assessed using receiver operating characteristic analysis. We additionally investigated the correlations of plasma Aβ ratios with amyloid-PET and CSF Alzheimer’s disease biomarkers, as well as platform agreement using Passing-Bablok regression and Bland-Altman analysis for both Aβ isoforms.

**Results:**

ELISA and SIMOA plasma Aβ_1–42_/Aβ_1–40_ detected cerebral amyloidosis with identical accuracy (ELISA: area under curve (AUC) 0.78, 95% CI 0.72–0.84; SIMOA: AUC 0.79, 95% CI 0.73–0.85), and both increased the performance of a basic demographic model including only age and *APOE-ε4* genotype (*p* ≤ 0.02). ELISA and SIMOA had positive predictive values of respectively 41% and 36% in cognitively normal elderly and negative predictive values all exceeding 88%. Plasma Aβ_1–42_/Aβ_1–40_ correlated similarly with amyloid-PET for both platforms (Spearman *ρ* = − 0.32, *p* <  0.0001), yet correlations with CSF Aβ_1–42_/t-tau were stronger for ELISA (*ρ* = 0.41, *p* = 0.002) than for SIMOA (*ρ* = 0.29, *p* = 0.03). Plasma Aβ levels demonstrated poor agreement between ELISA and SIMOA with concentrations of both Aβ_1–42_ and Aβ_1–40_ measured by SIMOA consistently underestimating those measured by ELISA.

**Conclusions:**

ELISA and SIMOA demonstrated equivalent performances in detecting cerebral amyloidosis through plasma Aβ_1–42_/Aβ_1–40_, both with high negative predictive values, making them equally suitable non-invasive prescreening tools for clinical trials by reducing the number of necessary PET scans for clinical trial recruitment.

**Trial registration:**

EudraCT 2009-014475-45 (registered on 23 Sept 2009) and EudraCT 2013-004671-12 (registered on 20 May 2014, https://www.clinicaltrialsregister.eu/ctr-search/trial/2013-004671-12/BE).

## Background

β-Amyloid (Aβ) and tau constitute key molecular hallmarks of Alzheimer’s disease (AD) and arise decades before cognitive symptoms. Their ensuing spread is associated with progressive neurodegeneration and cognitive decline [1–3]. In order to maximise the therapeutic window of slowing down neuronal loss and preventing cognitive decline, clinical trials in AD are shifting towards recruitment of nondemented individuals, including cognitively normal participants with increased cerebral Aβ [4]. To this end, surrogate biomarkers for amyloid pathology enable participant inclusion in early AD stages and prevent high screen failure rates. PET- and cerebrospinal fluid (CSF)-based amyloid biomarkers have proven to be valuable in the diagnosis of AD across the entire AD continuum. However, the high costs and limited availability of PET, and the invasive nature of both PET and CSF, render these methods impractical for large-scale screening imperative to clinical trial recruitment [5–7].

Alternatively, prescreening using less invasive and less expensive blood-based assays would streamline subject recruitment by reducing the required number of highly accurate amyloid-PET scans to verify cerebral amyloidosis before entering clinical trials [8]. Initially, classical ELISAs failed to accurately detect AD, making them unsuitable for implementation in prescreening [9, 10]. In response, ultrasensitive single molecule array (SIMOA) technology was introduced, enabling detection of cerebral amyloidosis through quantification of plasma amyloid ratios [11–13].

In parallel, improved ELISA formats have been developed, with promising clinical performances [14, 15]. Currently, head-to-head comparison in a large clinically, biochemically and radiologically well-characterised cohort is lacking, and between-study comparison of the clinical performances of the ELISA and SIMOA platform is hampered by the dependence of various performance parameters on inherent properties of the study design. Hence, no evidence to date favours one platform over the other. This is important as SIMOA assays require, for example, additional investment in dedicated instrumentation, whereas ELISAs do not. In this study, we concurrently quantified Aβ isoforms in plasma using commercially available EUROIMMUN ELISAs as well as SIMOA Amyblood assays employing identical antibody pairs. This allows a more accurate comparison of their clinical performances and consequently of their value in prescreening.

As a primary objective, we assessed and compared the abilities of the platforms to accurately detect cerebral amyloidosis by quantification of plasma Aβ_1–42_/Aβ_1–40_. In light of the interconnected pathophysiological pathways involving amyloid pathology and tau in AD, we additionally analysed the plasma ratios of respectively ELISA and SIMOA Aβ_1–42_ with ELISA total tau (t-tau). In CSF, the Aβ_1–42_/t-tau ratio outperforms the amyloid ratio in terms of predicting high risk profiles for progression in the AD continuum [9, 16]. In addition, an earlier study by our laboratory showed that, in CN subjects, CSF Aβ_1–42_/t-tau detects amyloid-PET positivity with higher accuracy than CSF Aβ_1–42_/Aβ_1–40_, especially when high specificity is required [17]. These findings provided the impetus for the investigation of its counterpart in plasma. Secondly, for each platform, correlations between plasma ratios and established AD biomarkers (i.e. amyloid-PET binding and CSF Aβ_1–42_/t-tau) were calculated. Finally, the agreement of Aβ measurements between platforms was assessed.

## Methods

### Study population

The study population consisted of 199 nondemented participants: 161 cognitively normal (CN) participants and 38 patients with amnestic mild cognitive impairment (aMCI). The two groups did not differ in age (*p* = 0.06), sex (*p* = 0.72) or years of education (*p =* 0.86). The CN participants stemmed from the Flemish Prevent AD Cohort KU Leuven (F-PACK), a larger longitudinal community-recruited study cohort of 180 CN elderly volunteers [18], preregistered under EudraCT 2009-014475-45 [19]. At inclusion, the F-PACK cohort was stratified for *APOE-ε4* genotype such that half of the included individuals carried at least one *APOE-ε4* allele [18, 20]. Among the F-PACK inclusion criteria, participants had to score within the normal range on detailed neuropsychological evaluation and have a Mini-Mental State Examination (MMSE) score of ≥ 27/30 and a Clinical Dementia Rating (CDR) scale score of 0. At baseline, participants underwent [^18^F]flutemetamol amyloid-PET and structural MRI. EDTA plasma samples at baseline, sampled between 2009 and 2016, were available for 165 F-PACK participants; however, four participants were excluded due to technical errors in the SIMOA Amyblood assays (coefficient of variation (CV) > 20%), yielding a CN subgroup of 161 participants.

aMCI patients (*n* = 38) stemmed from a consecutive academic memory clinic recruited longitudinal observational cohort, the *Biomarker-based adaptive development in Alzheimer’s disease* (BioAdaptAD) cohort, preregistered under EudraCT 2013-004671-12 [19]. All aMCI patients were recruited from the Memory Clinic of the University Hospitals Leuven. Among the BioAdaptAD inclusion criteria, participants had to be clinically followed with a current clinical diagnosis of aMCI. The aMCI participants all had unknown amyloid-PET or CSF status at the time of inclusion in the BioAdaptAD study. Clinical disease duration was on average 4.5 ± 3.2 years. Following the BioAdaptAD study protocol, aMCI participants received a [^18^F]florbetaben amyloid-PET scan, a structural MRI, EDTA blood sampling between 2015 and 2016 and detailed neuropsychological assessment.

### Amyloid-PET imaging

All participants underwent amyloid-PET on a 16-slice Biograph PET/CT scanner (Siemens, Erlangen, Germany) and structural MRI on a 3-T Achieva scanner (Philips, Best, The Netherlands), with the exception of one CN subject and three aMCI patients who had contraindications for MRI. For the latter four subjects, the mean MRI images calculated from amyloid-PET negative subjects of the respective cohorts were used for segmentation and calculation of the deformation field used in normalising the PET data. PET measurements were acquired in a 90- to 120-min post-injection window, and the standardised uptake value ratio was calculated in a composite volume of interest (SUVR_comp_) using participant-specific cerebellar grey matter as a reference region [18]. Amyloid-PET positivity was defined as a SUVR_comp_ above predefined cut-offs equal to 1.38 for [^18^F]flutemetamol PET [21] and 1.29 for [^18^F]florbetaben PET. For calculation of these cut-offs, we used the same methodology as the one employed in a previous study [22]. For both tracers, SUVR_comp_ values were converted to Centiloid (CL) values to allow correlation between cerebral amyloid burden and plasma biomarkers across the CN and aMCI subgroups (see Appendix 1).

Intermediate amyloid burden was defined as CL values between 20 and 50 and high amyloid burden as CL ≥ 50 [23]. Twenty-two (14%) CN participants showed intermediate amyloid burden (CL range 22.2–47.8), while eight (5%) showed high amyloid burden (CL range 66.25–184.9). aMCI patients generally had a higher prevalence of amyloid-PET positivity than CN participants, with four patients (11%) showing intermediate amyloid burden (CL range 27.0–36.9) and nine (24%) showing high amyloid burden (CL range 51.5–103.1).

### Cerebrospinal fluid assays

CSF samples were available for a subset of both subgroups (37 CN, 19 aMCI). In both subgroups, a lumbar puncture was performed with a 22G traumatic needle between L3/L4 and L4/L5. The CSF samples of CN participants were processed according to the F-PACK protocol; the collected CSF was transferred to a PP tube (Greiner Bio-One, 82050-278), followed by centrifugation at 1264*g* at 4 °C and aliquotation in 1.5-mL low-binding PP tubes (Kartell, 298). The CSF samples of aMCI patients were collected within the multicentre BioAdaptAD study, which adhered to a similar protocol; the collected CSF was transferred to a PP tube (Sarstedt, 62.610.018), followed by centrifugation for 10 min at 3000*g* at RT and aliquotation in 1.5-mL low-binding PP cryovials (Sarstedt, 72.703). The low-binding PP tubes were then placed on dry ice. Finally, all samples of both subgroups were stored at − 80 °C within 2 h after sampling. CSF Aβ_1–42_ and t-tau levels were determined by means of INNOTEST ELISAs (Fujirebio, Ghent, Belgium). In line with the International Working Group (IWG)-2 criteria, which commends combined analysis of CSF Aβ_1–42_ and p-tau or t-tau, we included CSF Aβ_1–42_/t-tau as a CSF-based AD biomarker.

### Plasma collection and processing

Blood was collected in K2EDTA-coated polyethylene terephthalate tubes (BD Diagnostics, BD367864). Samples of CN participants were processed according to the F-PACK study protocol, starting with centrifugation at 1200*g* for 10 min at 4 °C, followed by transfer of supernatant to polypropylene (PP) cryovials (Thermo Fisher Scientific, 363401, 500 μL plasma per tube) and subsequent storage at − 20 °C for 24 h before moving them to − 80 °C. aMCI patient samples were collected within the multicentre BioAdaptAD study, which adhered to a different protocol; samples were first centrifuged at 3000*g* for 15 min with subsequent division of the supernatant into PP cryovials (Sarstedt, 72.703) stored at − 80 °C within 2 h after sampling.

### Assay characteristics

We quantified EDTA plasma Aβ_1–40_ and Aβ_1–42_ with commercially available ELISA kits (EUROIMMUN, Lübeck, Germany), as well as with prototype SIMOA Amyblood assays (UMC Amsterdam and ADx NeuroSciences), which use the same sets of monoclonal antibodies: the 3D6 antibody, which is an N-terminal antibody that binds to residues 1–5 of the Aβ peptide, was used as the detector antibody and the C-terminal antibodies 21F12 and 2G3 were used as capture antibodies to capture respectively plasma Aβ_x–42_ and Aβ_x–40_ (Table 1). This differs from the singleplex and 3-Plex SIMOA assays (Quanterix, Lexington, MA, USA) employing the 6E10 antibody as a capture and detector antibody, respectively. The 6E10 antibody does not specifically target the N-terminus, but instead binds an RHD sequence located at residues 5–7 of the Aβ peptide [11, 12]. As a result, these SIMOA assays detect amyloid fragments of various lengths (Aβ_x–42_ and Aβ_x–40_) [24]. The Quanterix SIMOA assays use a different C-terminal antibody for Aβ_x–42_ (H31L21), but the same C-terminal antibody for Aβ_x–40_ as used in the SIMOA Amyblood assays and EUROIMMUN ELISAs (Thijssen, under review [25]).

**Table 1:** Analytical assay characteristics

	Platform	ELISA colorimetric			SIMOA	
	Analyte	Aβ_1–42_	Aβ_1–40_	t-tau	Aβ_1–42_	Aβ_1–40_
**Assay**	**Provider**	EUROIMMUN	EUROIMMUN	ADx	ADx	ADx
	**Catalogue number**	EQ 6521-9601	EQ 6511-9601	NA	NA	NA
	**Biofluid**	EDTA plasma	EDTA plasma	EDTA plasma	EDTA plasma	EDTA plasma
	**Status**	Commercial	Commercial	Prototype	Prototype	Prototype
	**Specificity**	Aβ_1–42_	Aβ_1–40_	6 tau isoforms	Aβ_1–42_	Aβ_1–40_
**Dilution**	**Pre-dilution factor**^**a**^	4	4	4	4	20
	**Final sample dilution**^**b**^	5	5	5	5.8	29
**Calibrator**	**Type**	Recombinant	Recombinant	Recombinant	Recombinant	Recombinant
	**No. of calibrator points**	7	7	7	7	7
	**Range, pg/mL**	1–40	1–75	1–100	1–64	1–64
**Patient samples**	**Number**	199	199	199	199	199
	**Range, pg/mL**	8.1–57.1	22.4–311.8	15.0–102.1	7.9–44.0	60.7–160.0
	**Within cal. range, %**	100%	100%	100%	100%	100%
**CV%**	**Intra-assay conc**	1.63	1.67	2.15	4.33	2.24
	**Inter-assay conc**	4.82	2.58	13.55	8.59	5.95
**Analytical sensitivity**	**LoD, pg/mL**	2.4	5.7	*Not determined*	0.46	0.77
	**LoQ, pg/mL**	3.5	12.5	*Not determined*	1.21	1.57
	**S/N ratio**	7.60	65.4	*Not determined*	11.0	240
**Antibodies** [26, 27]	**Name capture**	ADx102 (21F12)	ADx103 (2G3)	ADx203	ADx102 (21F12)	ADx103 (2G3)
	**Epitope capture (AA)**	Aβ_34–42_	Aβ_33–40_	Tau_194–204_	Aβ_34–42_	Aβ_33–40_
	**Name detector**	2G3	2G3	ADx204	2G3	2G3
	**Epitope detector (AA)**	Aβ_1–5_	Aβ_1–5_	Tau_N-terminus_	Aβ_1–5_	Aβ_1–5_
**Assay protocol**	**Incubation times, h**	3–0.5–0.5	3–0.5–0.5	3–0.5–0.5	2–0.08	2–0.08
	**Incubation T, °C**	18–25	18–25	18–25	18–25	18–25
	**Curve fit**	4PL	4PL	4PL	4PL	4PL
**QC panel, pg/mL**	**QC1: high**	30.5	189.2	22.3	13.7	83.2
	**QC2: intermediate**	26.6	149.1	18.9	19.7	75.9
	**QC3: intermediate**	22.0	116.8	21.7	13.4	69.7
	**QC4: low**	20.7	107.3	23.7	10.5	33.3
	**QC5: low spiked**	14.4	0.0	NA	21.6	21.8
	**QC6: high spiked**	117.9	125.6	NA	173.7	181.2
	**C1: low kit control**	23.3	114.6	NA	NA	NA
	**C2: high kit control**	45.1	186.9	NA	NA	NA

*AA* amino acid, *Aβ* β-amyloid, *CV* coefficient of variation, *LoD* limit of detection, *LoQ* limit of quantification, *S/N ratio* signal to noise ratio, *QC* quality control, *T* temperature, *t-tau* total tau

^a^Sample pre-dilution was performed using assay diluent in polypropylene low-binding 96-well microplates

^b^Final sample dilution during sample incubation step in assay protocol

EDTA plasma t-tau was quantified with a prototype ELISA designed by ADx NeuroSciences, which included an N-terminal detector antibody and a capture antibody targeting residues 194–204 of the tau protein (Table 1).

### Plasma amyloid and tau measurements

EUROIMMUN ELISA assays were performed manually according to the manufacturer’s protocol, and absorbance spectra were obtained with the CLARIOstar Plus microplate reader (BMG Labtech, Ortenberg, Germany). The lyophilized calibrators of multiple ELISA kits from the same lot were first reconstituted and then pooled per Aβ isoform in order to standardise the calibrator material among the different ELISA kits used. Subsequently, the reconstituted calibrators were aliquoted in separate PP tubes (Qiagen, 19560) per ELISA plate and stored at − 20 °C until testing. SIMOA Amyblood assays were performed as described earlier [28], and in-house developed ready-to-use calibrators were employed, which were composed of the same recombinant proteins (rPeptide, Athens, USA) as the ELISA calibrators.

The prototype ELISA for plasma t-tau included in-house developed ready-to-use calibrators constituted of recombinant t-tau protein (rPeptide, Athens, USA). No SIMOA-based quantification of plasma t-tau was performed. Consequently, the SIMOA-based Aβ_1–42_/t-tau ratio is a combination of the SIMOA Aβ_1–42_ measure and the ELISA t-tau measure.

The quality control (QC) panel was identical in all assays and was selected from a collection of 30 plasma samples donated by CN volunteers other than those in the F-PACK cohort. QC selection aimed at identifying one sample with consistently high levels of both amyloid isoforms (QC1), two samples with intermediate levels (QC2/QC3) and one sample with consistently low levels (QC4) of both amyloid isoforms when quantified by means of EUROIMMUN ELISA. For amyloid immunoassays, two additional QC samples were included consisting of an in-house prepared buffer spiked with respectively low (QC5) and high concentrations (QC6) of both recombinant Aβ_1–40_ and Aβ_1–42_ peptides identical to those used in the calibrators. Subsequently, all QCs were divided into 150-μL aliquots in PP vials (Sarstedt, 730.105) and stored at − 80 °C so that one vial was available for every ELISA and Amyblood run. The QCs provided by the EUROIMMUN ELISA kit (C1–2) were also reported (Table 1). No SIMOA-specific QC samples were available. It was observed that the Aβ_1–42_ concentration in the intermediate control sample QC2 was lower than in the high control sample QC1 when measured with ELISA, while it was higher when measured with the SIMOA assay. Of note, the QC panel was selected based on ELISA data and not SIMOA data. Moreover, the Aβ_1–42_ concentrations in the low, intermediate and high QC samples are all within a relatively close range, presumably because they all stemmed from CN volunteers. This, in addition to the substantial measurement difference in terms of values generated between the two platforms, is thought to cause the between-platform discrepancy in Aβ_1–42_ concentrations within the QC1 and QC2 sample.

Within all assays, plasma samples were randomised for analyses and all samples were analysed in duplicate within a total of four runs in four consecutive days. No correction for inter-assay variation was required, as inter-assay CVs were all below 15% (mean 7.10, range 2.58–13.55). Every vial was subjected to only one freeze/thaw cycle. All measured concentrations exceeded the limits of detection (LoDs) and limits of quantification (LoQs) and fell within the calibration ranges of the respective assays. The time interval between blood collection and measurement of plasma biomarkers was longer for the CN subgroup (median 6.51, IQR 5.36–7.78 years) than for the aMCI subgroup (median 3.24, IQR 3.04–3.83 years) (*p* <  0.0001), but did not differ between amyloid-PET negative (amyloid-PET−ve) and amyloid-PET positive (amyloid-PET+ve) participants within either subgroup (all *p >* 0.74*).*

### Statistical analyses

Statistical analyses were performed using GraphPad Prism 8.4.2 (GraphPad Software Inc., La Jolla, CA, USA) and MedCalc 19.0.3 (MedCalc, Ostend, Belgium) software. Normality was assessed with D’Agostino-Pearson test. Demographic continuous variables were compared between amyloid-PET groups with unpaired *t* tests or Mann-Whitney *U* tests in case of two groups, depending on normality, and with Kruskall-Wallis tests in case of three or more groups. Contingency tables were analysed by means of *χ*^2^ tests for categorical variables at a significance level of 0.05. Correlations between demographic variables and plasma biomarkers were assessed within the full nondemented cohort as well as in the CN and aMCI subgroups. Bonferroni correction was applied to adjust for multiple comparisons with two separate immunoassay platforms (ELISA and SIMOA, Bonferroni correction: α = 0.05/*k* compared platforms, *k* = 2, α = 0.03). In order to derive effect sizes for plasma levels depending on amyloid status, robust *d* values were calculated using the R package “WRS2” in R statistical software, version 3.6.2 (2019-12-12) (The R Foundation for Statistical Computing, https://www.r-project.org/). Robust *d* values are an alternative to Cohen’s standardised mean difference effect size [29] and do not assume a normal distribution of variables.

As primary outcome analysis, the performance of plasma Aβ_1–42_/Aβ_1–40_ to detect cerebral amyloidosis was compared between the ELISA and SIMOA platform using receiver operating characteristic (ROC) analyses for detecting amyloid-PET positivity based on binary classification of SUVR_comp_ values in the full nondemented cohort as well as in the subgroups (CN and aMCI, respectively). The areas under the ROC curve (AUCs) with 95% CIs were reported as measures of performance. Sensitivities, specificities, positive predictive values (PPVs) and negative predictive values (NPVs) were calculated for optimal cut-offs at maximised Youden index. For all biomarkers and their ratios, this AUC was compared to the AUC value adjusted for age and *APOE-ε4* genotype. To obtain this adjusted AUC value with 95% CIs, we first calculated a binary logistic regression model with amyloid-PET positivity as binary dependent variable and the plasma biomarker as well as age and *APOE-ε4* genotype as independent variables. In a next step, the result of this binary logistic regression, i.e. predicted probabilities, was entered in a ROC analysis to obtain the final adjusted AUC value. *APOE-ε4* genotype was specified by means of a dummy variable (non-carrier = 0, heterozygous carrier = 1, homozygous carrier = 2). Adjusted AUCs were only reported if they significantly differed from the unadjusted AUC. In addition, the adjusted AUCs were compared to the AUC of a basic demographic model, including only age and *APOE-ε4* genotype as independent variables, but no plasma biomarker or plasma biomarker ratio. Pairwise comparisons between ROC curves were performed with the DeLong method [30].

As a second objective, the correspondence of plasma biomarkers versus established AD biomarkers was assessed using Spearman rank correlations for ELISA and SIMOA measurements of plasma Aβ_1–42_/Aβ_1–40_ and Aβ_1–42_/t-tau ratios with (i) continuous Centiloid values as a measure for amyloid-PET binding and (ii) CSF Aβ_1–42_/t-tau. The latter contained data of a subgroup of cases for whom CSF samples were available (*n* = 56).

As a final objective, we examined the agreement of plasma amyloid measurements (commutability) between platforms in the entire nondemented study cohort (*n* = 199) using Mann-Whitney *U* tests to assess differences in median plasma Aβ measurements, Spearman rank correlations and Passing-Bablok regression analyses. The difference between the two assays is also shown graphically using non-parametric percentile Bland-Altman bias plots for which, by definition, the *Y* axis represents the difference between the two immunoassay platforms and the *X* axis represents the average of these measures. This allows the assessment of whether one method consistently under- or overestimates measurements of the same variable as compared to the other method.

## Results

Demographics and plasma biomarker data of all participants within the entire nondemented study population as well as within the CN and aMCI subgroups are shown in total, as well as stratified by amyloid-PET status in Table 2. In the total group of nondemented participants (*p* = 0.006), but not in subgroups (*p* > 0.19), amyloid-PET+ve individuals were generally older than amyloid-PET−ve individuals. No differences in sex distribution or education were found between amyloid-PET groups in either subgroup (all *p* > 0.54). The amyloid-PET+ve group had a higher proportion of *APOE-ε4* carriers only within the CN subgroup (*p* = 0.03). The lower proportion of *APOE-ε4* carriers in the aMCI subgroup compared to the CN subgroup (*p* = 0.03) is a direct consequence of the recruitment strategy (see the “Methods” section).

**Table 2:** Characteristics of the study population and subgroups in total and stratified by amyloid-PET status

	Study population			CN subgroup			aMCI subgroup		
	Total	Aβ−	Aβ+	Total	Aβ−	Aβ+	Total	Aβ−	Aβ+
**Number (%)**	199	161 (81)	38 (19)	161	137 (85)	24 (15)	38	24 (63)	14 (37)
**Mean age (SD), years**	70 (6)	69 (6)	72 (5)^b^	69 (6)	69 (6)	71 (5)	71 (7)	69 (6)	74 (6)
**Female,** ***n*** **(%)**	89 (45)	72 (45)	17 (45)	73 (45)	61 (45)	12 (50)	16 (42)	11 (46)	5 (36)
**Mean education (SD), years**	14 (3)	14 (3)	14 (4)	14 (3)	14 (3)	14 (4)	14 (4)	14 (4)	14 (3)
***APOE-ε4*** **carriers/homozygous,** ***n (*%)**	92/7 (46)	70/5 (43)	22/2 (58)	82/6 (51)	65/5 (47)	17/1 (71)^a^	10/1 (26)	5/0 (21)	5/1 (36)
**Median MMSE (IQR), /30**	29 (2)	29 (2)	29 (2)^a^	29 (2)	29 (2)	29 (2)	29 (2)	29 (1)	27 (3)^a^
**Median ELISA plasma Aβ**_**1–42**_**/Aβ**_**1–40**_ **(IQR)**	0.17 (0.03)	0.18 (0.03)	0.15 (0.02)^d^	0.18 (0.03)	0.18 (0.03)	0.15 (0.02)^d^	0.17 (0.02)	0.18 (0.03)	0.16 (0.01)^b^
**Median ELISA plasma Aβ**_**1–42**_**/t-tau (IQR)**	1.23 (0.50)	1.32 (0.49)	1.00 (0.29)^d^	1.25 (0.49)	1.29 (0.49)	1.02 (0.34)^c^	1.21 (0.58)	1.41 (0.51)	0.89 (0.27)^d^
**Median SIMOA plasma Aβ**_**1–42**_**/Aβ**_**1–40**_ **(IQR)**	0.26 (0.06)	0.26 (0.06)	0.21 (0.05)^d^	0.26 (0.06)	0.26 (0.05)	0.21 (0.05)^d^	0.24 (0.07)	0.28 (0.06)	0.22 (0.03)^c^
**Median SIMOA plasma Aβ**_**1–42**_**/t-tau (IQR)**	0.93 (0.41)	0.99 (0.39)	0.73 (0.24)^d^	0.94 (0.37)	0.99 (0.38)	0.74 (0.27)^c^	0.92 (0.45)	1.14 (0.33)	0.70 (0.21)^d^

*aMCI* amnestic mild cognitive impairment, *Aβ* β-amyloid, *CDR* Clinical Dementia Rating, *CN* cognitively normal, *ELISA* enzyme-linked immunosorbent assay, *IQR* interquartile range, *MMSE* Mini-Mental State Examination, *SIMOA* single molecule array, *t-tau* total tau. *p* values reflect comparisons between Aβ−ve and Aβ+ve groups: ^a^*p* <  0.05; ^b^*p* <  0.01; ^c^*p* <  0.001; ^d^*p* <  0.0001

In the total cohort of nondemented individuals (*p* = 0.02), as well as in the aMCI subgroup (*p* = 0.04), amyloid-PET+ve subjects had lower MMSE scores than amyloid-PET−ve subjects. MMSE scores did not differ between amyloid-PET groups within the CN subgroup (*p* = 0.44).

Plasma Aβ_1–42_/Aβ_1–40_ was lower in amyloid-PET+ve subjects than in amyloid-PET−ve subjects for both the ELISA (*d* = 1.17) and SIMOA (*d* = 1.24) platforms in the total nondemented cohort as well as in the CN (ELISA *d* = 1.25; SIMOA *d* = 1.03*)* and aMCI subgroup (ELISA *d* = 1.097; SIMOA *d* = 1.44, all *p* ≤ 0.01) (see supplementary Figure 1a). The same was true for plasma Aβ_1–42_/t-tau (*d >* 0.73, all *p* ≤ 0.0006) (see supplementary Figure 1b).

Neither plasma amyloid nor t-tau was influenced by sex (*p* > 0.14) or years of education (all *p* > 0.30) for either platform. ELISA plasma Aβ_1–40_ (*ρ* = 0.29, *p* <  0.0001), Aβ_1–42_ (*ρ* = 0.19, *p* = 0.006) and t-tau (*ρ* = 0.39, *p* <  0.0001) as well as SIMOA plasma Aβ_1–40_ (*ρ* = 0.36, *p* <  0.0001) and Aβ_1–42_ (*ρ* = 0.18, *p* = 0.01) were weakly positively correlated with age.

### Comparison between the performance of ELISA and SIMOA biomarkers to detect cerebral amyloidosis

As primary outcome analysis, we compared the EUROIMMUN ELISA and SIMOA Amyblood platform with respect to their ability to determine cerebral amyloidosis on PET through quantification of plasma Aβ_1–42_/Aβ_1–40_.

**Fig. 1:**
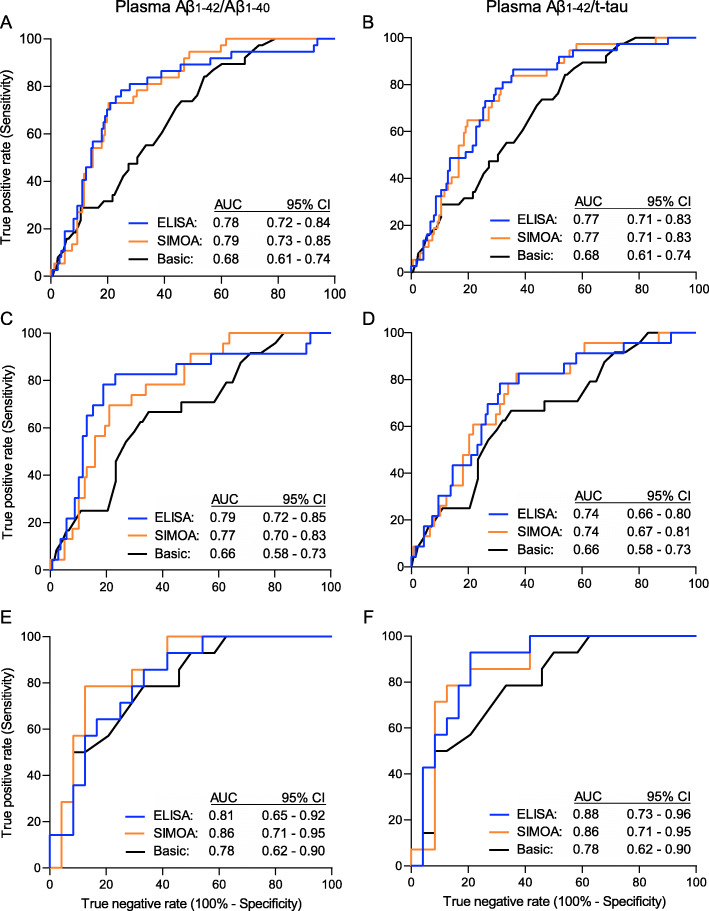
ROC curves of plasma Aβ_1–42_/Aβ_1–40_ and Aβ_1–42_/t-tau to detect cerebral amyloidosis: ELISA versus SIMOA. ROC curves of plasma Aβ_1–42_/Aβ_1–40_ (left) and Aβ_1–42_/t-tau (right) are shown with amyloid-PET status as the standard-of-truth in the entire study population (*n* = 199) (**a**, **b**) as well as in the CN (*n* = 161) (**c**, **d**) and aMCI subgroup (*n* = 38) (**e**, **f**), when Aβ isoforms were measured with either ELISA (blue) or SIMOA assays (orange). Note that the AUCs for ELISA and SIMOA are based on plasma biomarker measurements on their own, without inclusion of age or *APOE-ε4* genotype in the model. Additionally, the ROC curve of the basic demographic model, including only age and *APOE-ε4* genotype, is shown (black) on each plot together with its corresponding AUC for the respective subgroups. Amyloid-PET positivity as binary input for ROC was defined as a SUVR_comp_ above a predefined cut-off of 1.38 for [^18^F]flutemetamol PET [21] and 1.29 for [^18^F]florbetaben PET. For calculation of these cut-offs, we used the same methodology as the one employed in a previous study [22]. aMCI, amnestic mild cognitive impairment; AUC, area under curve; Aβ, β-amyloid; CI, confidence interval; CN, cognitively normal; ROC, receiver operating characteristic; SIMOA, single molecule array; t-tau, total tau

The discriminative performance of ELISA Aβ_1–42_/Aβ_1–40_, as indicated by ROC AUCs, did not differ from that of the SIMOA platform in the total nondemented study population (Fig. 1a, *p* = 0.85) nor in the CN (Fig. 1c, *p* = 0.81) or the aMCI (Fig. 1e, *p* = 0.58) subgroup (see supplementary Table 1). Furthermore, high similarity between ELISA and SIMOA with respect to sensitivity, specificity, PPV and NPV was observed at optimal Youden index associated cut-offs, with the exception of plasma Aβ_1–42_/Aβ_1–40_ in the aMCI subgroup, which yielded higher specificities when measured with SIMOA. NPVs reached high values (all ≥ 88%) for both platforms (Table 3).

**Table 3:** Optimal plasma biomarker cut-offs with corresponding performance parameters for detecting amyloid-PET positivity

Plasma ratio	Platform	Group	Cut-off	Sensitivity, %	Specificity, %	PPV, %	NPV, %
**Age,** ***APOE-ε4***		**Total**	na	84 (69–94)	46 (38–54)	27 (23–31)	93 (85–96)
		**CN**	na	67 (45–84)	65 (56–73)	25 (19–33)	92 (86–95)
		**aMCI**	na	79 (49–95)	67 (45–84)	58 (43–72)	84 (65–94)
**Aβ**_**1–42**_**/Aβ**_**1–40**_	**ELISA**	**Total**	< 0.159	78 (62–90)	75 (68–82)	42 (34–50)	93 (88–96)
		**CN**	< 0.159	78 (56–93)	81 (74–87)	41 (32–51)	96 (91–98)
		**aMCI**	< 0.170	86 (57–98)	67 (45–84)	60 (45–73)	89 (68–97)
	**SIMOA**	**Total**	< 0.230	74 (57–87)	80 (72–86)	46 (37–55)	93 (88–96)
		**CN**	< 0.229	70 (47–87)	79 (71–86)	36 (27–46)	94 (89–97)
		**aMCI**	< 0.226	79 (49–95)	88 (68–97)	79 (55–92)	88 (71–95)
**Aβ**_**1–42**_**/t-tau**	**ELISA**	**Total**	< 1.19	84 (68–94)	64 (56–71)	35 (30–41)	95 (89–97)
		**CN**	< 1.12	78 (56–93)	69 (60–76)	30 (23–37)	96 (90–98)
		**aMCI**	< 1.18	93 (66–100)	79 (58–93)	72 (54–85)	95 (74–99)
	**SIMOA**	**Total**	< 0.862	79 (63–90)	68 (60–75)	36 (30–43)	93 (88–96)
		**CN**	< 0.899	83 (61–95)	63 (54–71)	27 (22–33)	96 (90–99)
		**aMCI**	< 0.815	79 (49–95)	88 (68–97)	79 (55–92)	88 (71–95)

*aMCI* amnestic mild cognitive impairment, *Aβ* β-amyloid, *CN* cognitively normal, *NPV* negative predictive value, *PPV* positive predictive value, *t-tau* total tau

Inclusion of plasma Aβ_1–42_/Aβ_1–40_ of either platform into a basic demographic model with age and *APOE-ε4* genotype improved the performance of the basic model in detecting amyloid-PET positivity (ELISA: *p* = 0.02; SIMOA: *p* = 0.0009; see supplementary Table 2) within the total study population. The performance of this adjusted model was identical to that of the unadjusted biomarker-only model in the total study population as well as in the subgroups (all *p* ≥ 0.32; see supplementary Table 2) and had similar sensitivities, specificities, NPVs and PPVs at highest Youden index (see supplementary Table 3).

Plasma Aβ_1–42_/t-tau yielded similar results: its discriminative performance did not differ when plasma Aβ_1–42_ was measured with either ELISA or SIMOA in the total nondemented study cohort (Fig. 1b) nor in the subgroups (Fig. 1d, f) (all *p* ≥ 0.76; see supplementary Table 1). Also sensitivities, specificities, NPVs and PPVs were similar between platforms (Table 3).

Plasma Aβ_1–42_/t-tau and Aβ_1–42_/Aβ_1–40_ had similar performances when amyloid isoforms were measured with either platform and within both subgroups; however, in the CN subgroup, plasma Aβ_1–42_/Aβ_1–40_ had higher specificities and PPVs than plasma Aβ_1–42_/t-tau for both ELISA and SIMOA. In contrast, within the aMCI subgroup, the specificities and PPVs did not differ between the two biomarker ratios for either platform (Table 3).

Inclusion of plasma Aβ_1–42_/t-tau into a basic demographic model with age and *APOE-ε4* genotype increased discriminative performance of the basic model in the total nondemented cohort (ELISA: *p* = 0.004; SIMOA: *p* = 0.003; see supplementary Table 2). The performances of these adjusted models were identical to those of the unadjusted biomarker-only models (all *p* ≥ 0.14; see supplementary Table 2) and had similar sensitivities, specificities, NPVs and PPVs at highest Youden index (see supplementary Table 3).

Plasma Aβ_1–42_ alone also identified amyloid-PET positivity, but only within the CN subgroup (*p* <  0.0001), and within the total study population, its performance was lower than that of the ratios (*p* ≤ 0.003; see supplementary Figure 2), regardless of the employed platform.

### Correlation of plasma biomarkers with amyloid imaging and CSF Aβ_1–42_/t-tau

As a secondary outcome analysis, we assessed the correlations between plasma biomarkers, on the one hand, and amyloid-PET and CSF Aβ_1–42_/t-tau, on the other hand, for each platform.

**Fig. 2:**
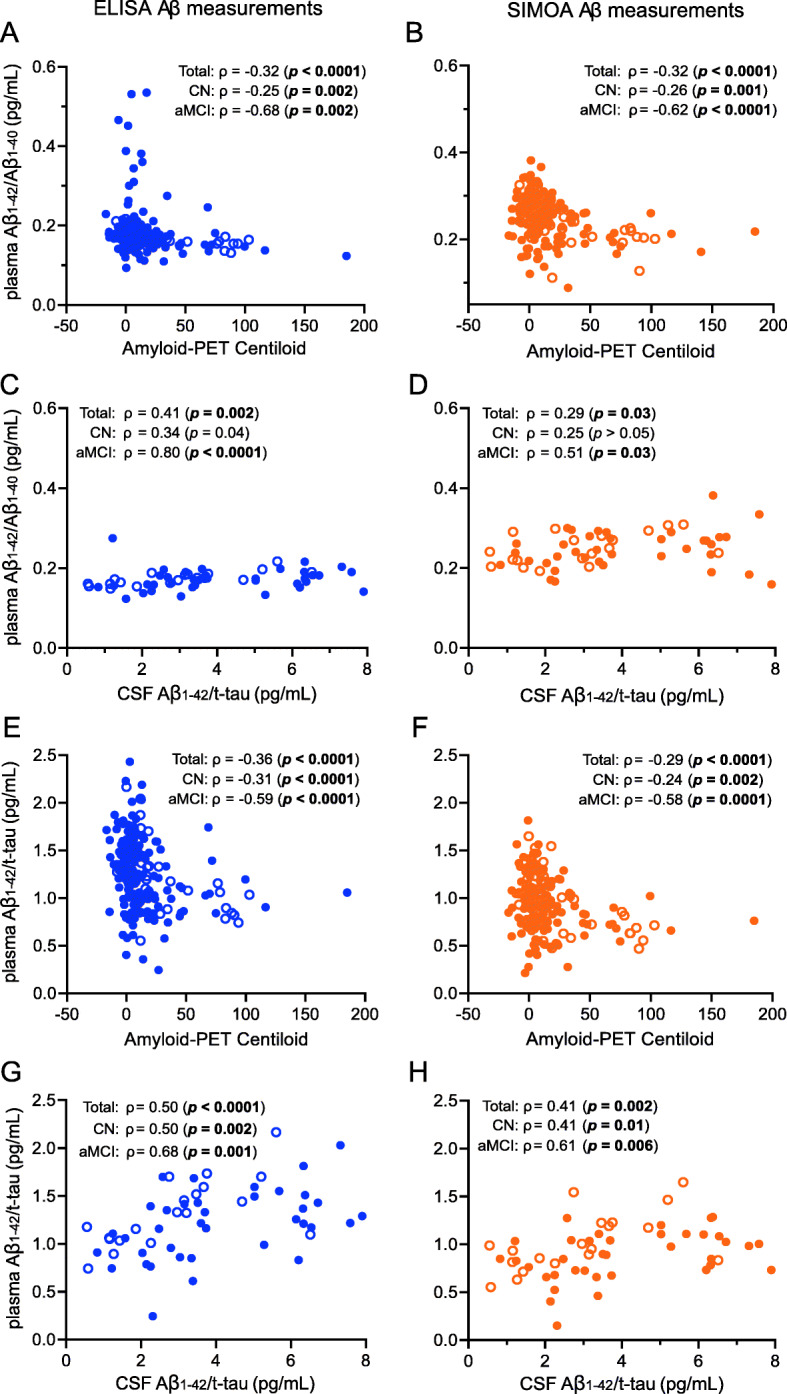
Correlations of ELISA and SIMOA plasma Aβ_1–42_/Aβ_1–40_ and Aβ_1–42_/t-tau with established PET- and CSF-based AD biomarkers. Plasma Aβ_1–42_/Aβ_1–40_ (**a**–**d**) and Aβ_1–42_/t-tau (**e**–**h**) with ELISA (left, blue) and SIMOA (right, orange) Aβ measurements were plotted against amyloid-PET binding (i.e. Centiloid values) (*n* = 199: 161 CN controls and 38 aMCI patients) and CSF Aβ_1–42_/t-tau (*n* = 56: 37 CN controls and 19 aMCI patients). Filled circles represent measurements in CN controls, and open circles represent measurements in aMCI patients. Spearman rank correlations were calculated for the entire study population as well as for the CN and aMCI subgroups. *p* values are indicated in bold when significant after correction for multiple comparisons (significance level α = 0.05/2 = 0.03). aMCI, amnestic mild cognitive impairment; Aβ, β-amyloid; CN, cognitively normal; ELISA, enzyme-linked immunosorbent assay; SIMOA, single molecule array; t-tau, total tau

For both platforms, plasma Aβ_1–42_/Aβ_1–40_ decreased as amyloid-PET binding increased. Correlations between plasma Aβ_1–42_/Aβ_1–40_ and amyloid-PET were also present within both subgroups, albeit stronger in the aMCI subgroup (Fig. 2a, b). In a subset of 56 subjects for whom CSF AD biomarkers were available, lower ELISA levels of plasma Aβ_1–42_/Aβ_1–40_ correlated with lower CSF Aβ_1–42_/t-tau levels in the total study population, as well as in the aMCI subgroup, but not in the CN subgroup (Fig. 2c). For SIMOA Aβ_1–42_/Aβ_1–40_, these correlations were also present, albeit weaker (Fig. 2d).

Plasma Aβ_1–42_/t-tau yielded similar results: for both platforms, the ratio decreased as amyloid-PET binding increased (Fig. 2e, f) and increased with rising CSF Aβ_1–42_/t-tau (Fig. 2g, h). However, in contrast to plasma Aβ_1–42_/Aβ_1–40_, this latter correlation was now also present within the CN subgroup.

With respect to individual biomarkers, plasma Aβ_1–42_ correlated with amyloid-PET within the aMCI subgroup for both platforms (ELISA: *p* = 0.03; SIMOA: *p* = 0.004; see supplementary Figure 3a-b) and with CSF Aβ_1–42_/t-tau when measured with ELISA in the CN subgroup (*p* = 0.004; see supplementary Figure 3f). No correlations between plasma Aβ_1–40_ and amyloid-PET or CSF Aβ_1–42_/t-tau were observed for either platform (all *p* > 0.05; see supplementary Figure 3c,d,h,i).

### Correlation and amyloid value agreement (commutability) between ELISA and SIMOA

**Fig. 3:**
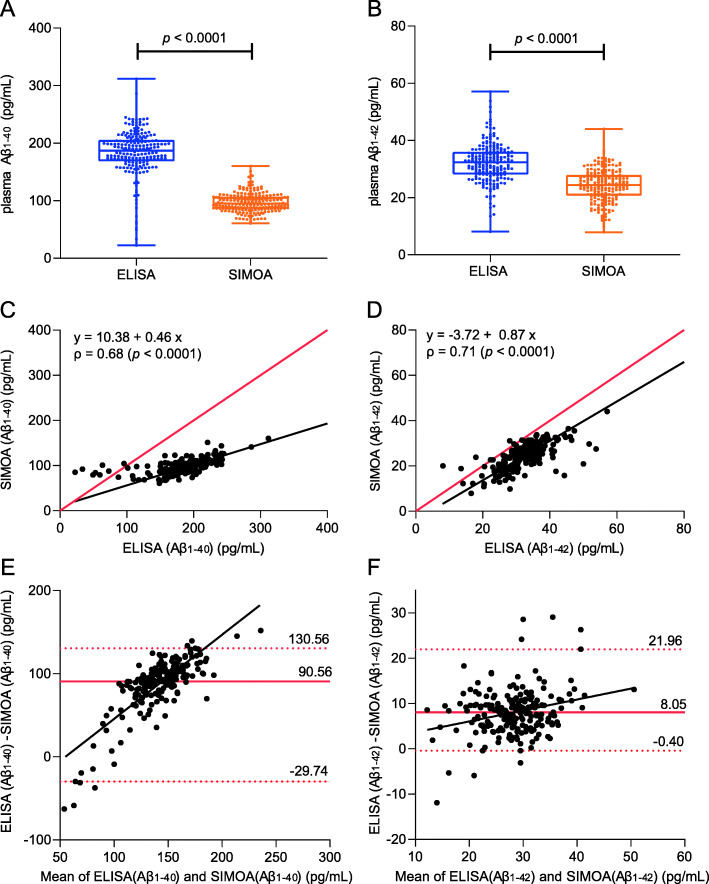
Correlations and commutability between ELISA and SIMOA measurements of β-amyloid (Aβ) isoforms Aβ_1–40_ and Aβ_1–42_. **a**, **b** Box and whisker plots of ELISA (left, blue) and SIMOA (right, orange) measurements of plasma Aβ_1–40_ (**a**) and Aβ_1–42_ (**b**) are shown. The middle line of the box represents the median. The lower and upper line represent, respectively, the first and third quartiles, and the whiskers represent the range. Individual data points are superimposed on the boxplot. Agreement between the two platforms is shown for both Aβ isoforms (**c**–**f**). Scatter plots and Passing-Bablok regression analysis of plasma Aβ_1–40_ (**c**) and Aβ_1–42_ (**d**) concentrations measured by SIMOA Amyblood in function of their concentrations measured by ELISA. The regression line is shown in black. Spearman rank correlations were calculated to assess the non-linear relationship between the two methods for both isoforms. **e**, **f** Non-parametric percentile method of Bland-Altman graphically shows the agreement between the two immunoassay platforms for respectively Aβ_1–40_ (**e**) and Aβ_1–42_ (**f**). The solid red line represents the median of differences between measurements of the two methods from the same subject. The upper and the lower red dashed lines represent respectively the 97.5th and 2.5th percentile of the measurement differences between which 95% of measurements is situated. Aβ, β-amyloid; ELISA, enzyme-linked immunosorbent assay; SIMOA, single molecule array

Lastly, we assessed the agreement of the plasma Aβ isoform measurements between platforms. Median Aβ_1–40_ (Fig. 3a) and median Aβ_1–42_ levels (Fig. 3b) were lower for SIMOA than for ELISA. For both Aβ isoforms, the range of plasma concentrations measured by ELISA was broader than that of SIMOA. Aβ measurements were strongly correlated between the assays, but showed poor agreement (i.e. low commutability), especially for Aβ_1–40_ (Fig. 3c, d). Further statistical modelling using Passing-Bablok regression revealed that SIMOA Amyblood Aβ_1–40_ levels were proportionally lower than ELISA Aβ_1–40_ levels as the regression slope was lower than 1 (slope 0.46, 95% CI 0.39 to 0.52; Fig. 3c). This large proportional difference was presumably driven by the fact that for ELISA Aβ_1–40_ measurements below 110 pg/mL, correlation with SIMOA measurements was lost, a so-called floor effect. This floor effect causes the data points to deviate from the linear Passing-Bablok regression curve at low concentration values (Fig. 3c). This observation is also evident graphically on the Bland-Altman plots, which showed poor commutability of Aβ_1–40_ measurements between assays in this lower range (Fig. 3e).

Plasma Aβ_1–42_ levels also showed proportional differences between assays, be it smaller in effect than Aβ_1–40_ (regression slope 0.87, 95% CI 0.78–0.98; Fig. 3d). However, in contrast to plasma Aβ_1–40_, plasma Aβ_1–42_ also showed a constant difference of − 3.72 pg/mL between assays (intercept = − 3.72, 95% CI − 7.14 to − 1.02; Fig. 3d). Despite smaller and more consistent average differences in plasma Aβ_1–42_ values between platforms across the concentration range (compared to plasma Aβ_1–40_), more overall variability was observed. This is evident as a broader data point distribution on the plasma Aβ_1–42_ Bland-Altman plot (Fig. 3f) compared to the plasma Aβ_1–40_ plot (Fig. 3e).

Furthermore, plasma ratios differed between platforms: ELISA measured lower plasma Aβ_1–42_/Aβ_1–40_ ratios and higher Aβ_1–42_/t-tau ratios than SIMOA in the entire study population (*p* <  0.0001, not shown) as well as within amyloid-PET stratified groups (see supplementary Figure 1a-b).

## Discussion

The current study demonstrates that the accuracy of determining amyloid-PET positivity in nondemented participants through measurement of plasma Aβ_1–42_/Aβ_1–40_ was similar for ELISA and SIMOA Amyblood assays. Moreover, we showed that inclusion of plasma Aβ_1–42_/Aβ_1–40_ in a basic demographic model including age and *APOE-ε4* genotype resulted in a higher discriminative performance than that of the basic demographic model alone. Furthermore, ELISA and SIMOA plasma Aβ_1–42_/Aβ_1–40_ measurements correlated to the same extent with amyloid-PET binding within the total study population as well as within both subgroups, and correlated with CSF Aβ_1–42_/t-tau in the aMCI subgroup, albeit weaker for SIMOA than for ELISA. The performance of plasma Aβ_1–42_/t-tau and its correlations with amyloid-PET and CSF Aβ_1–42_/t-tau were also similar when Aβ_1–42_ was measured with either ELISA or SIMOA and were comparable with what was observed for plasma Aβ_1–42_/Aβ_1–40_.

When comparing our findings to other studies employing SIMOA-based amyloid assays, of note is that the novel SIMOA Amyblood assays used here differ from the previously used Quanterix SIMOA assays [11, 12] with respect to the amyloid isoforms detected by the employed antibodies. More specifically, while our respective Amyblood assays detect the intact Aβ_1–42_ and Aβ_1–40_ peptides, the Quanterix assays do not bind the Aβ isoforms at their N-terminus. Consequently, they detect peptides of various lengths including not only the intact Aβ peptide but also the N-terminus fragmented (Aβ_x–42_ or Aβ_x–40_) peptides. An early study employing the first singleplex Quanterix SIMOA assays for plasma Aβ_x–42_ and Aβ_x–40_ in a large cohort encompassing the full AD continuum reported a lower age- and sex-adjusted AUC (0.62) for plasma Aβ_x–42_/Aβ_x–40_ [11]. Of note, the combination of the detector-capture antibody pair within these singleplex Quanterix assays opposes the one of the SIMOA Amyblood assays. Specifically, in these so-called first-generation SIMOA assays, the detector antibody was directed against the C-terminus and thus isoform-specific, while the capture antibody binds both Aβ isoforms. This not only prevents multiplexing, but also results in a lower assay specificity. In the Quanterix SIMOA Neurology 3-Plex kit, the antibody pair combination was reversed, thus matching the orientation of the Amyblood assays. A study in a smaller subjective cognitive decline (SCD) population employing the 3-Plex kit reported a higher age- and *APOE-ε4* genotype-adjusted AUC (0.79) than in the first study, which employed the singleplex assays. This value was highly similar to the unadjusted AUC obtained in the CN subgroup of the current study (ELISA, 0.79; SIMOA, 0.77), yet the unadjusted AUC they obtained was lower (0.68) [12]. Another group, which developed their own immunoassay on the SIMOA platform, also intended to detect the intact Aβ_1–42_ and Aβ_1–40_ peptides and reported an unadjusted AUC of 0.79 for plasma Aβ_1–40_/Aβ_1–42_ in a SCD population slightly larger than the current study sample [13]. This is similar to the unadjusted AUC we observed for plasma Aβ_1–42_/Aβ_1–40_ measured with ELISA as well as SIMOA in our total study population and CN subgroup. Besides, similar to the current study, the AUC did not improve upon correction for age and *APOE-ε4* genotype. In conjunction with the present findings, this suggests that detecting Aβ isoforms specifically and in their entirety, rather than a mixture of different Aβ isoform-derived fragments, results in better assay performance. However, this needs to be confirmed through head-to-head comparison.

Recently, alternative ELISA assays, i.e. *ABtest42* and *ABtest40* (Araclon Biotech Ltd., Zaragoza, Spain), were developed in which plasma was pre-treated to disrupt interactions between Aβ peptides and other plasma components. This allows measurement of free as well as bound Aβ concentrations, the so-called total plasma Aβ levels. In contrast, SIMOA or traditional ELISAs only measure peptides that are free in plasma. An early study using *ABtests* to measure total plasma Aβ_1–42_/Aβ_1–40_ in CN participants reported an age- and *APOE-ε4* genotype-adjusted AUC comparable to our unadjusted AUC (0.79) [14]. A larger follow-up study, additionally correcting for sex, showed a slightly increased corrected AUC (0.86), which was higher than the AUC of a basic demographic model including only age, sex and *APOE-ε4* genotype [15]. The translatability of these studies to our findings is limited, since no unadjusted AUCs were reported.

Although first-generation ELISAs failed to accurately detect AD—causing the field to focus on alternative quantification methods—the clinical performance of the novel ELISAs has improved and, as our findings indicate, is no longer inferior to the clinical performance of the ultrasensitive SIMOA platform. This evolution is attributable to various technological advancements in the immunoassay field. First, the EUROIMMUN ELISAs used in the current study and the *ABtests* used previously [14, 15] employ C- and N-terminal antibodies [31], while previous ELISAs did not, and consequently detected different Aβ fragments [10, 32]. Secondly, the evolution of the assay design and the improved conjugation method in the novel ELISAs might have resulted in improved sensitivities, thus enabling the detection of more subtle variations in biomarker profiles than attainable with first-generation ELISAs [10, 32]. However, this hypothesis can only be substantiated through a head-to-head comparison of the different ELISAs. Lastly, the increased understanding of the important influence of pre-analytical variables on Aβ measurements has motivated the standardisation and optimisation of pre-analytical sample handling, which results in more reliable outputs [33].

Lastly, immunoprecipitation coupled with mass spectrometry (IP-MS) methods have also been developed to quantify Aβ species in blood. Two IP-MS studies, including healthy elderly participants as well as MCI and AD dementia patients, showed unadjusted AUCs of respectively 0.84 (95% CI 0.79–0.89) for plasma Aβ_1–40_/Aβ_1–42_ [34] and 0.89 for plasma Aβ_x–42_/Aβ_x–40_ [35]. Another recent IP-MS study including earlier AD stages, better resembling our nondemented study population, demonstrated an unadjusted AUC of 0.87 (95% CI 0.80–0.94) for plasma Aβ_x–42_/Aβ_x–40_ [36]. These AUC values are slightly higher than what was observed in our total nondemented study population or in previous studies employing ELISA or SIMOA assays in healthy subjects, but strongly resemble what we obtained in our aMCI subgroup. However, considering the substantial variations in study design, no strong statements concerning the methods’ superiority can be made before head-to-head comparisons are performed.

The discrepancies in clinical performance between platforms in different studies could be guided by underlying differences in the study design, such as cohort size, participant selection, prevalence of amyloid-PET positivity or employed standard-of-truth rather than clinically relevant differences. For example, most studies employing IP-MS methodology [34, 35] include a wide AD spectrum instead of exclusively CN volunteers, which naturally results in a higher performance. Some studies use only one amyloid-PET tracer [11, 13, 15], while others use a mix of different amyloid-PET tracers [12, 14, 34, 36]; however, a previous study demonstrated better correspondence between amyloid-PET and plasma Aβ_1–40_/Aβ_1–42_ when using exclusively [^11^C]PiB PET as standard-of-truth compared to a mix of [^18^F]-labelled tracers [34]. Another study used a combination of amyloid-PET and CSF Aβ_42_ as standard-of-truth [35], the latter being less specific to detect early AD compared to CSF ratios or amyloid-PET [16], which could have possibly confounded the performance of the investigated plasma biomarker. Besides, while one study stratified the study population based on visual reads of amyloid-PET [12], others used (semi-)quantitative measures such as SUVRs [11, 13–15, 34, 36] or binding potential [35]. Furthermore, some studies find an influence of covariates, such as age and *APOE-ε4* genotype, on biomarker performance [12, 34, 36], while others, including ours, did not [13]. This further illustrates the importance of a head-to-head comparison of analytical platforms, as performed in the current study, which allows a direct assessment of their respective utilities for prescreening in clinical trials.

In addition to plasma Aβ_1–42_/Aβ_1–40_, we also examined the clinical utility of plasma Aβ_1–42_/t-tau to detect cerebral amyloidosis. We hypothesised that this would be a promising candidate as it reflects the multidimensionality of AD by capturing both amyloidosis and tau-related neurodegeneration. As mentioned in the introduction, the potential of a combined marker has already been shown in CSF. Besides, plasma t-tau/Aβ_1–42_ detects tau deposition with good accuracy in a cohort of predominantly CN subjects, but also including MCI and demented patients [37]. In the current study, plasma Aβ_1–42_/t-tau, just like plasma Aβ_1–42_/Aβ_1–40_, outperformed plasma Aβ_1–42_ alone_._ This increased performance of the ratios has been linked to the correction for interindividual differences in total Aβ production in Aβ_1–42_/Aβ_1–40_ and might result from capturing multiple AD dimensions in Aβ_1–42_/t-tau. Besides, the reduction in analytical confounding factors that typically arise in protein analyses, such as the presence of various blood cells, might also play an important role in the increased performance [38]. The lower specificity and PPV of plasma Aβ_1–42_/t-tau compared to plasma Aβ_1–42_/Aβ_1–40_ in the CN subgroup but not in the aMCI subgroup might argue for the use of plasma Aβ_1–42_/Aβ_1–40_ in prescreening of CN subjects, while plasma Aβ_1–42_/t-tau is a potential alternative in later AD stages.

Our results imply that ELISA and SIMOA could substantially and to a similar degree reduce the amount of amyloid-PET scans necessary to recruit a pre-set number of healthy amyloid-PET+ve participants. Within our study population, around 15% of CN subjects was classified as amyloid-PET+ve, which is similar to what was observed in other studies using amyloid-PET imaging in CN subjects [39]. Based on this prevalence, one would need to screen approximately 6667 CN participants in order to recruit 1000 individuals with evidence of amyloid burden on PET. Based on the PPVs in our study, prescreening with blood-based tests on the ELISA platform would reduce this number with 63% for Aβ_1–42_/Aβ_1–40_ and with 50% for Aβ_1–42_/t-tau. For the SIMOA platform, the reductions are slightly smaller, namely 58% for Aβ_1–42_/Aβ_1–40_ and 44% for Aβ_1–42_/t-tau. In comparison, prescreening of CN participants solely based on age and *APOE-ε4* genotype would reduce the number of necessary PET scans by only 40%. Previous studies using IP-MS [36] or *ABtest* ELISAs [14, 15] to quantify the plasma amyloid ratio reported higher PPVs than the ones observed here, which might give the impression that these methods are superior to the SIMOA or conventional ELISA technology. However, the prevalence of amyloid-PET positivity was also higher in these other studies (≈ 30%), which directly influences the reported PPV. Ultimately, the reported number of saved amyloid-PET scans ranged between 53 and 62%, which is in line with our results. Moreover, we found strong NPVs up to 96% for both platforms, indicating that up to 96% of participants excluded through blood-based prescreening were amyloid-PET−ve, thereby strongly increasing the prevalence of amyloid-PET positivity in the group subjected to further examination.

Despite similar clinical performances, plasma Aβ levels demonstrated poor agreement between the platforms, especially for Aβ_1–40_ in the low detection range. More specifically, concentrations of both plasma Aβ_1–42_ and Aβ_1–40_ measured by SIMOA consistently underestimated those measured by ELISA, which was most pronounced for plasma Aβ_1–40_. Consequently, also the plasma biomarker ratios differed between platforms. The floor effect of SIMOA Aβ_1–40_ concentrations in plasma samples with ELISA Aβ_1–40_ concentrations below 110 pg/mL was not expected given the lower LoQ for the SIMOA Amyblood assay than for the ELISA assay, albeit both are well below 110 pg/mL (1.57 and 12.5 pg/mL, respectively). This warrants further investigation. The poor between-platform agreement in measured values emphasises that caution is required in the interpretation of plasma biomarker values when employing different platforms and argues in favour of assay harmonisation or at least a consistent platform use in clinical trials. This is especially important when applying cut-offs, which can differ significantly between platforms given the difference in absolute values measured by each specific assay. Besides, the range of Aβ measurements was much higher for ELISA than for SIMOA. Unlike for CSF Aβ_1–42_ [40], no certified reference material has been developed for Aβ isoforms in plasma. This prevents harmonisation of diagnostic assays through calibration of their Aβ levels. Therefore, no certain statements can be made about the reliability of Aβ measurements reported in the current or previous studies until such reference material is available. However, the strong correlations between plasma ratios measured by the two platforms and their equally accurate performance in detecting cerebral amyloidosis indicate they are both valuable in clinical research settings. Moreover, intra-assay and inter-assay CVs were all below respectively 5% (mean 2.40, range 1.63–4.33) and 15% (mean 7.10, range 2.58–13.55), indicating high precision for all employed assays.

### Limitations

We acknowledge some potential limitations in our study design. The aMCI subgroup had low prevalence of amyloid-PET positivity. This can be explained by the fact that the aMCI subgroup also included participants who had been cognitively stable for multiple years. Further, the cross-sectional study design warrants confirmation of our findings with longitudinal data and neuropathological findings. From a technical perspective, with regard to plasma Aβ_1–42_/t-tau, this ratio was not purely SIMOA-based but a combination of SIMOA (Aβ_1–42_) and ELISA (t-tau) technology. Moreover, we did not compare the ELISA and SIMOA measurements to mass spectrometry, which is considered to be an absolute measurement method. Lastly, the centrifugation parameters within the plasma and CSF processing protocols differed between the CN and aMCI cohort. While they do not greatly influence biomarker levels in CSF, they are critical during plasma processing as platelets are important sources of Aβ and tau [41–43]. However, this discrepancy does not influence our primary study objective, namely the performance comparison between platforms, since every sample is measured in parallel on both platforms.

## Conclusions

Overall, despite poor between-platform agreement in amyloid measurements, the clinical performances of the ELISA and SIMOA platform were comparable for plasma Aβ_1–42_/Aβ_1–40_ as well as for plasma Aβ_1–42_/t-tau. These findings have important implications for the scalability of plasma Aβ measures as ELISA is currently much more widely available than SIMOA. In the context of participant recruitment for clinical trials, SIMOA Amyblood did not provide additional benefit to commercially available ELISA assays in this nondemented study population. Consequently, both plasma Aβ platforms would reduce the current high screen failure rate and optimise the cost and efficiency of clinical trials for AD in a similar way.

## Supplementary Information

**Additional file 1: Appendix 1.** Conversion of [^**18**^F]flutemetamol and [^**18**^F]florbetaben SUVR to the Centiloid scale. **Supplementary Table 1.** Plasma amyloid and tau performance (AUC) differences between platforms and subgroups for detecting amyloid-PET positivity. **Supplementary Table 2.** Performance of adjusted biomarker models relative to a basic demographic model and unadjusted biomarker models **Supplementary Table 3.** Sensitivities, specificities, PPVs and NPVs of logistic regression models corrected for age and *APOE-ε4* genotype. **Supplementary Figure 1.** Comparison of ELISA and SIMOA measurements of plasma Aβ_1-42_/Aβ_1-40_ and Aβ_1-42_/t-tau. **Supplementary Figure 2.** ROC curves of plasma amyloid and tau with amyloid-PET as the standard-of-truth. **Supplementary Figure 3.** Correlations of ELISA and SIMOA plasma amyloid and tau levels with Centiloids and CSF Aβ_1-42_/t-tau.

## Data Availability

The datasets used and/or analysed during the current study are available from the corresponding author on reasonable request.

## Abbreviations

AD: Alzheimer’s disease
aMCI: Amnestic mild cognitive impairment
Amyloid-PET+ve: Amyloid-PET positive
Amyloid-PET−ve: Amyloid-PET negative
AUC: Area under the curve
Aβ: β-Amyloid
BioAdaptAD: Biomarker-based adaptive development in Alzheimer’s disease
CDR: Clinical Dementia Rating
CSF: Cerebrospinal fluid
CV: Coefficient of variation
CL: Centiloid
CN: Cognitively normal
F-PACK: Flemish Prevent AD Cohort KU Leuven
IP-MS: Immunoprecipitation coupled with mass spectrometry
LoD: Limit of detection
LoQ: Limit of quantification
MMSE: Mini-Mental State Examination
NPV: Negative predictive value
PP: Polypropylene
PPV: Positive predictive value
QC: Quality control
ROC: Receiver operating characteristic
SCD: Subjective cognitive decline
SIMOA: Single molecule array
SUVR_comp_: Composite standardised uptake value ratio
t-tau: Total tau
