# Supplementary material for: Comparison of ELISA- and SIMOA-based quantification of plasma Aβ ratios for early detection of cerebral amyloidosis
Source: Alzheimers Res Ther. 2020 Dec 5;12:162. doi: 10.1186/s13195-020-00728-w (PMC7719262; doi:10.1186/s13195-020-00728-w)
Supplement: Supplementary file 1 — Additional file 1: Appendix 1. Conversion of [18F]flutemetamol and [18F]florbetaben SUVR to the Centiloid scale. Supplementary Table 1. Plasma amyloid and tau performance (AUC) differences between platforms and subgroups for detecting amyloid-PET positivity. Supplementary Table 2. Performance of adjusted biomarker models relative to a basic demographic model and unadjusted biomarker models Supplementary Table 3. Sensitivities, specificities, PPVs and NPVs of logistic regression models corrected for age and APOE-ε4 genotype. Supplementary Figure 1. Comparison of ELISA and SIMOA measurements of plasma Aβ1-42/Aβ1-40 and Aβ1-42/t-tau. Supplementary Figure 2. ROC curves of plasma amyloid and tau with amyloid-PET as the standard-of-truth. Supplementary Figure 3. Correlations of ELISA and SIMOA plasma amyloid and tau levels with Centiloids and CSF Aβ1-42/t-tau. [file 13195_2020_728_MOESM1_ESM.docx]

**Appendix 1. Conversion of [^18^F]flutemetamol and [^18^F]florbetaben SUVR to the Centiloid scale**

To allow quantification of the uptake of both tracers on a common scale, standardized uptake value ratios (SUVRs) of the respective tracers were converted to Centiloids (CLs). First, level-1 Centiloid analysis was replicated; ^11^C-Pittsburgh Compound B (^11^C-PiB) PET and MRI images from 34 young controls (YCs) and 45 Alzheimer’s disease (AD) patients, which were downloaded from the Global Alzheimer’s Association Interactive Network (GAAIN) website [1], were preprocessed according to the standard CL method described previously [2]. This allowed the establishment of the 0 CL (^PiB^SUVR_YC-0_) and 100 CL (^PiB^SUVR_AD-100_) anchor points. The obtained CL values of the entire cohort were compared to the ones published in the original CL paper [2] through linear regression analysis. Next, we performed level-2 processing using two previously described reference cohorts, also available on the GAAIN website, consisting of 50 AD patients and 22 YCs that underwent MRI, [^11^C]PiB PET and [^18^F]flutemetamol ([^18^F]FMM) PET [3], and 25 elderly subjects and 10 YCs who underwent MRI, [^11^C]PiB PET and [^18^F]florbetaben ([^18^F]FBB) PET [4]. PET scans were available as summed images of 4 x 5min rebinned frames acquired in the 50-70min time window after tracer injection for [^11^C]PiB PET, and the 90-110min time window after tracer injection for [^18^F]FMM and [^18^F]FBB PET. After downloading, the [^11^C]PiB PET scans of both reference cohorts [3,4] were processed with the standard CL method using SPM8 software, while the [^18^F]FMM and [^18^F]FBB PET scans of the same subjects were processed according to an in-house developed pipeline using SPM12 software as described above. Calibration of [^18^F]FMM SUVR (^FMM^SUVR) and [^18^F]FBB PET SUVR (^FBB^SUVR) to the CL scale is a two-step process. First, linear regression between ^PiB^SUVRs calculated with the standard CL method and respectively ^FMM^SUVRs and ^FBB^SUVRs calculated with our own preprocessing pipeline yielded a slope *m* and an intercept *b* which were used to calculate ^PiB-Calc^SUVR values, so that:

$${}^{FMM/FBB}{SUVR=m \times{}^{PiB}{SUVR+b}}$$

$${}^{PiB-Calc}{SUVR}= \frac{{}^{FMM/FBB}{SUVR-b}}{m}$$

Next, CLs could be calculated using the ^PiB-Calc^SUVR values and the 0- and 100-anchor points obtained through level-1 analysis, so that:

$${}^{FMM/FBB}{CL}=100 \times\frac{{}^{PiB-Calc}{SUVR}- {}^{PiB}{SU{VR}_{YC-0}}}{{}^{PiB}{SU{VR}_{AD-100}- {}^{PiB}{SU{VR}_{YC-0}}}}$$

Through linear regression between the SUVR values and the calculated CL values, the following conversion formulas were obtained:${}{CL=127.6 \times{}{SUVR-149}}$ for [^18^F]FMM and ${}{CL=147 \times{}{SUVR-166.5}}$ for [^18^F]FBB. Repeating the processing of the PET images from our own study cohort using SPM12 software, now utilising the 90-110 minute time window instead of the 90-120 minute time window, allowed us to use this conversion formula for the calculation of CLs for all study subjects.

Linear regression between our own calculated [^11^C]PiB CL values and the ones published in the original CL paper yielded an R^2^ of 0.9993, a slope of 0.9987 and an intercept of 0.1313, all within the accepted ranges (R^2^ > 0.98, a slope between 0.98 and 1.02, an intercept between -2 and 2) (A). Linear regression between ^PiB^SUVR values calculated with the standard CL method and respectively ^FMM^SUVR and ^FBB^SUVR values calculated with our own pipeline showed good agreement between the original method and our own method for both tracers with R^2^ = 0.9311 for [^18^F]FMM (B) and R^2^ = 0.9632 for [^18^F]FBB (C) both well above the recommended threshold of 0.7. The relative variance – defined by the ratio of the standard deviation (SD) of CL values calculated in the YCs of the reference cohorts (7.12 for ^FMM^CL and 5.44 for ^FBB^CL) to the SD of the CL values calculated in the YCs in the cohort described in the original CL publication (4.41) – was equal to 1.64 for [^18^F]FMM and 1.23 for [^18^F]FBB PET.


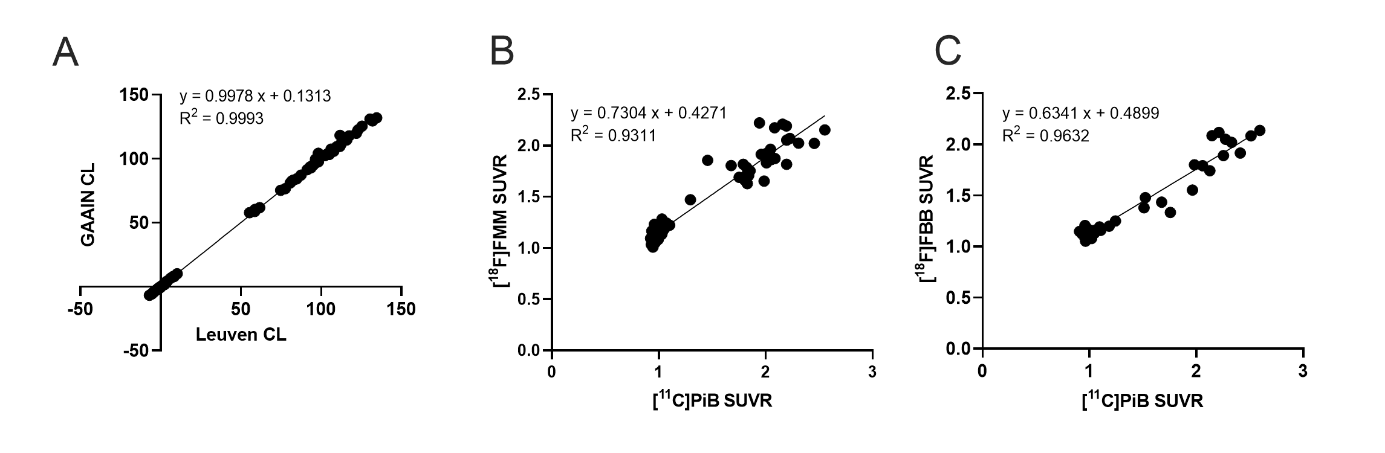


Supplementary Table 1. Plasma amyloid and tau performance (AUC) differences between platforms and subgroups for detecting amyloid-PET positivity

|  |  | **Study population** |  | **CN subgroup** |  | **aMCI subgroup** | ***p(GROUP)*** |
| --- | --- | --- | --- | --- | --- | --- | --- |
| **Aβ_1-42_/Aβ_1-40_** | **ELISA** | 0.78 (0.72-0.84) |  | 0.79 (0.72-0.85) |  | 0.81 (0.65-0.92) | 0.77 |
|  | **SIMOA** | 0.79 (0.73-0.85) |  | 0.77 (0.70-0.83) |  | 0.86 (0.71-0.95) | 0.26 |
|  | ***p(PLATFORM)*** | *0.85* |  | *0.81* |  | *0.58* |  |
| **Aβ_1-42_/t-tau** | **ELISA** | 0.77 (0.71-0.83) |  | 0.74 (0.66-0.80) |  | 0.88 (0.73-0.96) | 0.08 |
|  | **SIMOA** | 0.77 (0.71-0.83) |  | 0.74 (0.67-0.81) |  | 0.86 (0.71-0.95) | 0.13 |
|  | ***p(PLATFORM)*** | *0.97* |  | *0.98* |  | *0.76* |  |
| **Aβ_1-42_** | **ELISA** | 0.63 (0.56-0.70) |  | 0.70 (0.62-0.77) |  | 0.66 (0.49-0.81) | 0.76 |
|  | **SIMOA** | 0.63 (0.56-0.70) |  | 0.69 (0.61-0.76) |  | 0.71 (0.53-0.84) | 0.85 |
|  | ***p(PLATFORM)*** | *0.97* |  | *0.90* |  | *0.55* |  |
| **Aβ_1-40_** | **ELISA** | 0.59 (0.51-0.65) |  | 0.55 (0.47-0.63) |  | 0.55 (0.38-0.71) | 0.97 |
|  | **SIMOA** | 0.61 (0.54-0.68) |  | 0.51 (0.43-0.60) |  | 0.68 (0.51-0.82) | 0.16 |
|  | ***p(PLATFORM)*** | *0.49* |  | *0.39* |  | *0.05* |  |
| **t-tau** | **ELISA** | 0.72 (0.65-0.78) |  | 0.64 (0.56-0.72) |  | 0.82 (0.66-0.92) | 0.05 |
|  | **SIMOA** | NA |  | NA |  | NA | NA |
|  | ***p(PLATFORM)*** | NA |  | NA |  | NA |  |

AUCs are given for all tested biomarkers with their 95% CIs*. p(GROUP)* represent the *p* value of the difference in biomarker performance between the CN and aMCI subgroups. *p(PLATFORM)* represents the *p* value of the difference in biomarker performance between the ELISA and SIMOA platform. aMCI, mild cognitive impairment; Aβ, β-amyloid; CN, cognitively normal; SIMOA, single molecule array; t-tau, total tau

**Supplementary Table 2. Performance of adjusted biomarker models relative to a basic demographic model and unadjusted biomarker models**

|  |  | | **Study population** | | |  | **CN subgroup** | | |  | **aMCI subgroup** | | |
| --- | --- | --- | --- | --- | --- | --- | --- | --- | --- | --- | --- | --- | --- |
|  |  | **AUC (CI)** | | ***p_1_*** | ***p_2_*** |  | **AUC (CI)** | ***p_1_*** | ***p_2_*** |  | **AUC (CI)** | ***p_1_*** | ***p_2_*** |
| **ELISA Aβ_1-42_/Aβ_1-40_** | | | 0.78 | ***0.02*** | *0.91* |  | 0.75 | *0.14* | *0.33* |  | 0.84 | 0.38 | 0.82 |
|  |  |  | (0.72-0.83) |  |  |  | (0.68-0.82) |  |  |  | (0.68-0.94) |  |  |
| **SIMOA Aβ_1-42_/Aβ_1-40_** | | | 0.81 | ***0.0009*** | *0.48* |  | 0.76 | *0.05* | *0.64* |  | 0.92 | 0.06 | 0.32 |
|  |  |  | (0.75-0.86) |  |  |  | (0.69-0.83) |  |  |  | (0.78-0.98) |  |  |
| **ELISA Aβ_1-42_/t-tau** | | | 0.79 | ***0.004*** | *0.22* |  | 0.75 | *0.12* | *0.35* |  | 0.88 | 0.16 | 1.00 |
|  |  |  | (0.73-0.85) |  |  |  | (0.67-0.81) |  |  |  | (0.73-0.96) |  |  |
| **SIMOA Aβ_1-42_/t-tau** | | | 0.80 | ***0.003*** | *0.14* |  | 0.77 | *0.08* | *0.16* |  | 0.89 | 0.10 | 0.62 |
|  |  |  | (0.74-0.85) |  |  |  | (0.70-0.83) |  |  |  | (0.74-0.97) |  |  |
| **ELISA Aβ_1-42_** | | | 0.70 | *0.69* | ***0.02*** |  | 0.74 | *0.12* | *0.26* |  | 0.83 | 0.23 | 0.06 |
|  |  |  | (0.61-0.74) |  |  |  | (0.67-0.81) |  |  |  | (0.68-0.93) |  |  |
| **SIMOA Aβ_1-42_** | | | 0.70 | *0.69* | ***0.02*** |  | 0.74 | *0.07* | *0.18* |  | 0.87 | 0.15 | 0.08 |
|  |  |  | (0.61-0.74) |  |  |  | (0.67-0.81) |  |  |  | (0.72-0.95) |  |  |
| **ELISA Aβ_1-40_** | | | 0.69 | *0.55* | ***0.009*** |  | 0.66 | *0.86* | *0.14* |  | 0.78 | 0.88 | 0.05 |
|  |  |  | (0.62-0.75) |  |  |  | (0.58-0.73) |  |  |  | (0.62-0.90) |  |  |
| **SIMOA Aβ_1-40_** | | | 0.69 | *0.47* | ***0.01*** |  | 0.66 | *0.62* | *0.06* |  | 0.80 | 0.81 | 0.10 |
|  |  |  | (0.62-0.75) |  |  |  | (0.58-0.73) |  |  |  | (0.63-0.91) |  |  |
| **ELISA t-tau** | | | 0.69 | *0.59* | ***0.02*** |  | 0.67 | *0.83* | *0.50* |  | 0.82 | 0.49 | 0.92 |
|  |  |  | (0.62-0.75) |  |  |  | (0.60-0.75) |  |  |  | (0.66-0.93) |  |  |
| **Base**  **(Age, *APOE-ε4*)** | | | 0.68 | NA | NA |  | 0.66 | NA | NA |  | 0.78 | NA | NA |
|  |  |  | (0.61-0.74) |  |  |  | (0.58-0.73) |  |  |  | (0.62-0.90) |  |  |

The age- and *APOE-ε4* genotype-adjusted AUC of the ROC curve of each plasma biomarker and their ratios were compared to the unadjusted AUCs as well as to the AUC of a basic demographic model. Unadjusted AUCs were calculated through ROC analysis of plasma biomarker levels in amyloid-PET+ve vs amyloid-PET-ve subjects. Adjusted AUCs were calculated by first calculating the binary logistic regression model with amyloid-PET positivity as binary dependent variable and the plasma biomarker as well as age and *APOE-ε4* genotype as independent variables. Entering the predicted probabilities of this model into a ROC analysis yielded the adjusted AUCs. The basic demographic model is a binary logistic regression model including only age and *APOE-ε4* genotype, but no plasma biomarker, and its AUC was calculated in the same way. Adjusted AUCs are given in the left column of every group. The p value (*p_1_*) of the difference between the adjusted AUC of each plasma biomarker and the AUC of the basic demographic model is given in the middle column of each group. The p value (*p_2_*) of the difference between the adjusted AUC and the unadjusted AUC of each plasma biomarker is given in the right column of each group.

Significant *p* values are indicated in bold. Bonferroni correction: significance level α = 0.05/2 = 0.03. aMCI, amnestic mild cognitive impairment; AUC, area under the receiver operating characteristic curve; Aβ, β-amyloid; CN, cognitively normal; SIMOA, single molecule array; t-tau, total tau

**Supplementary Table 3. Sensitivities, specificities, PPVs and NPVs of logistic regression models corrected for age and *APOE-ε4*** **genotype**

|  |  | **Study sample** | | | |  | **CN subgroup** | | | |  | **aMCI subgroup** | | | |
| --- | --- | --- | --- | --- | --- | --- | --- | --- | --- | --- | --- | --- | --- | --- | --- |
|  |  | **%Sens** | **%Spec** | **PPV** | **NPV** |  | **%Sens** | **%Spec** | **PPV** | **NPV** |  | **%Sens** | **%Spec** | **PPV** | **NPV** |
| **ELISA Aβ_1-42_/Aβ_1-40_** | | 76 | 69 | 30 | 94 |  | 75 | 65 | 29 | 94 |  | 71 | 87 | 50 | 95 |
| **SIMOA Aβ_1-42_/Aβ_1-40_** | | 79 | 75 | 35 | 95 |  | 75 | 69 | 30 | 94 |  | 93 | 87 | 57 | 99 |
| **ELISA Aβ_1-42_/t-tau** | | 74 | 82 | 42 | 95 |  | 71 | 74 | 33 | 94 |  | 93 | 83 | 50 | 98 |
| **SIMOA Aβ_1-42_/t-tau** | | 84 | 71 | 34 | 96 |  | 75 | 69 | 31 | 94 |  | 93 | 83 | 50 | 99 |
| **Basic** |  | 84 | 46 | 27 | 93 |  | 67 | 65 | 25 | 92 |  | 79 | 67 | 58 | 84 |
| **(age, *APOE-ε4*)** | |  |  |  |  |  |  |  |  |  |  |  |  |  |  |

Sensitivities and specificities for detecting amyloid-PET status are given for plasma Aβ_1-42_/Aβ_1-40_ and Aβ_1-42_/t-tau cut-offs corresponding to the highest Youden index for the ELISA and SIMOA platform in the total nondemented study cohort as well as in the CN and aMCI subgroups, separately. aMCI, amnestic mild cognitive impairment; Aβ, β-amyloid; CN, cognitively normal; SIMOA, single molecule array; t-tau, total tau

**Supplementary Figure 1. Comparison of ELISA and SIMOA measurements of plasma Aβ_1-42_/Aβ_1-40_ and Aβ_1-42_/t-tau**

**
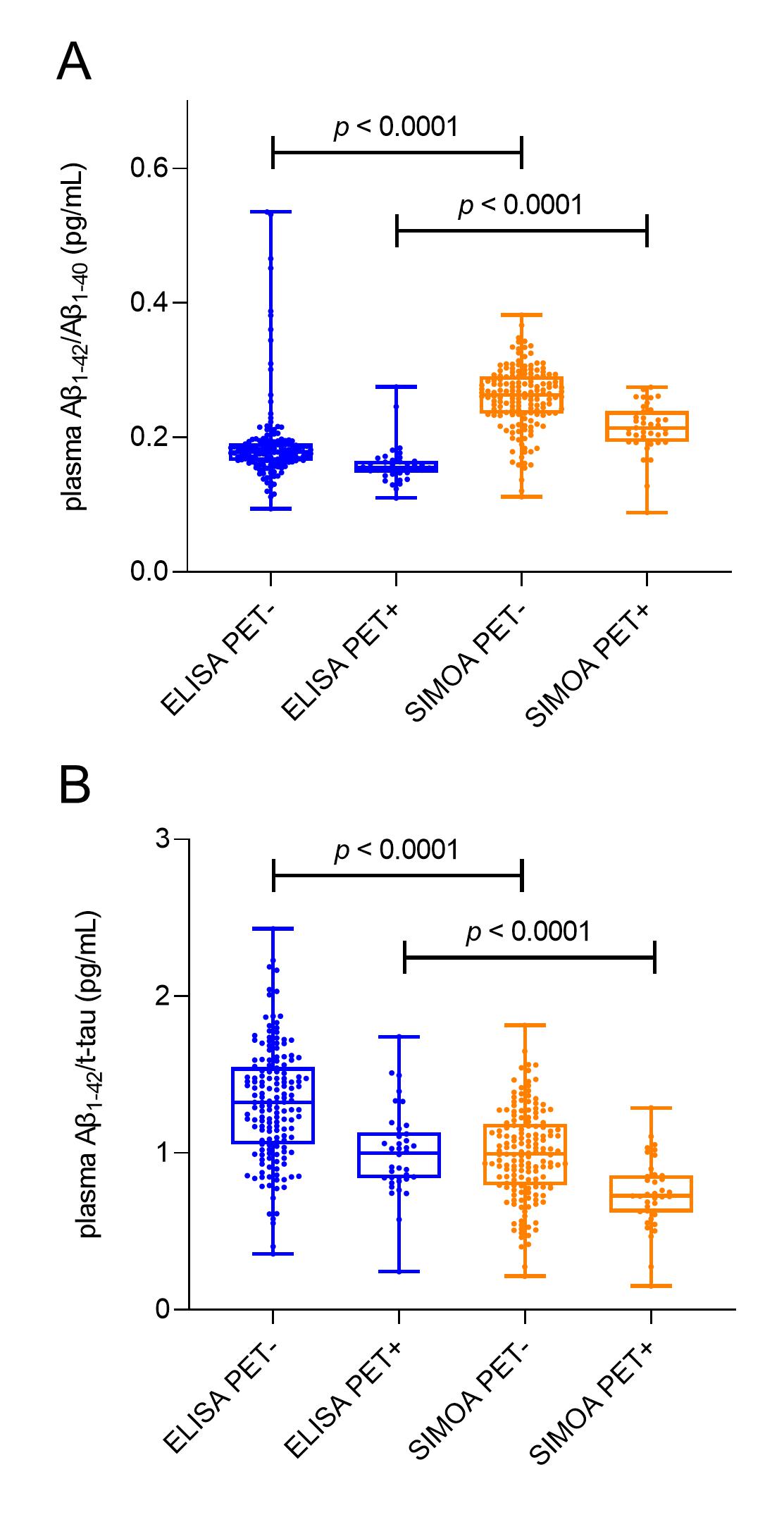
**

Box and whisker plots of ELISA (left, blue) and SIMOA (right, orange) measurements stratified for amyloid-PET status of plasma Aβ_1-42_/Aβ_1-40_ (A) and Aβ_1-42_/t-tau (B) are shown. The middle line of the box represents the median. The lower and upper line represent, respectively, the first and third quartiles and the whiskers represent the range. Individual data points are superimposed on the boxplot. Aβ, β-amyloid; ELISA, enzyme-linked immunosorbent assay; SIMOA, single molecule array; t-tau, total tau

**Supplementary Figure 2. ROC curves of plasma amyloid and tau with amyloid-PET as the standard-of-truth**

**
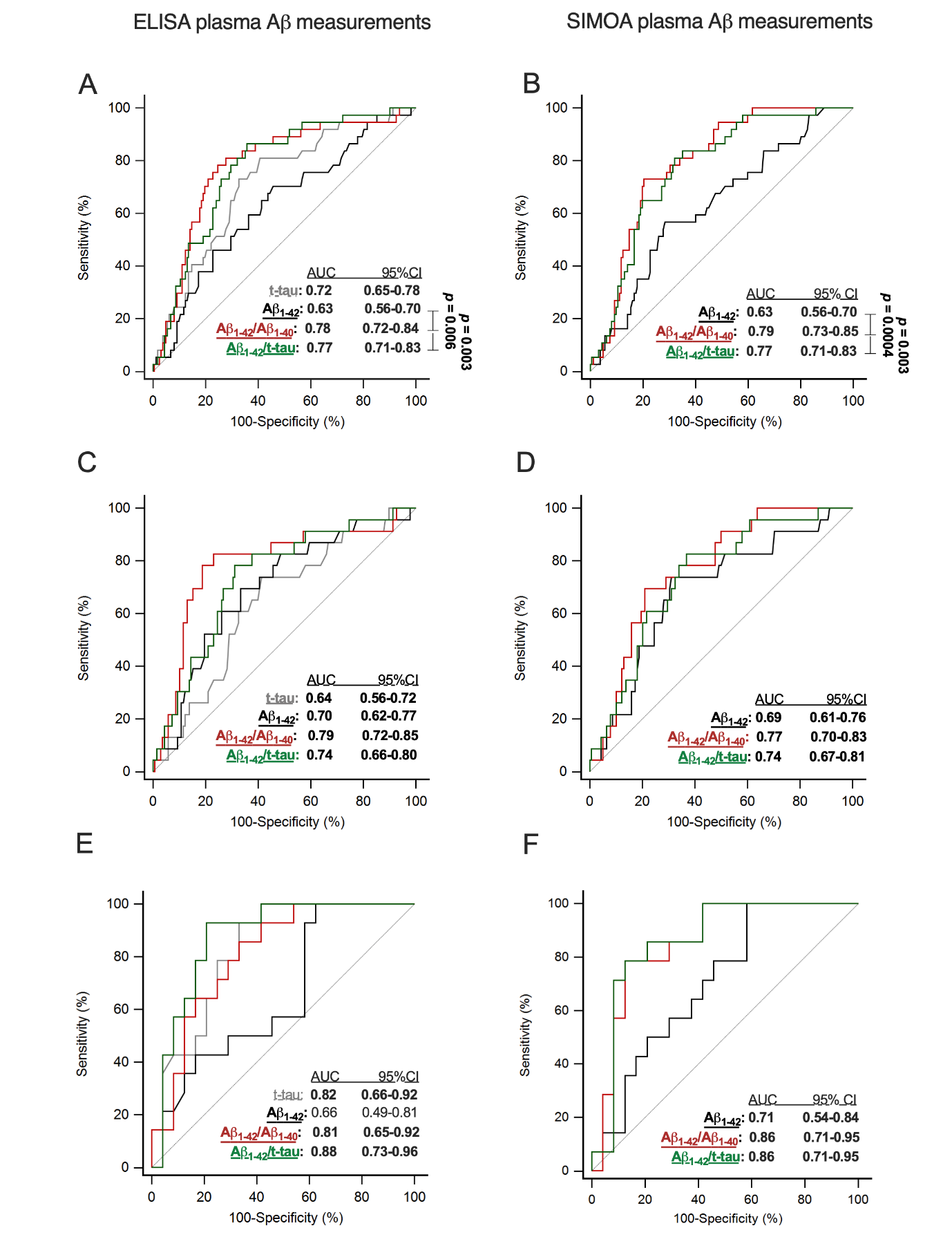
**

ROC curves of plasma t-tau, Aβ_1-42_, Aβ_1-42_/Aβ_1-40_ and Aβ_1-42_/t-tau are shown with amyloid-PET status as the standard of truth and this within the total study population (n = 199) (A-B) as well as in the separate CN (n = 161) (C-D) and aMCI subgroups (n = 38) (E-F) when Aβ isoforms were measured with either ELISA (left) or SIMOA (right). Amyloid-PET positivity was defined as a SUVR_comp_ above a predefined cut-off (1.38 for [^18^F]flutemetamol PET^7^ and 1.29 for [^18^F]florbetaben PET, calculated as described previously^8^). The colours of the curves correspond to the biomarkers as indicated in the legend. Pairwise comparisons between AUCs of the tested biomarkers within each subgroup and tested with the same immunoassay platform were performed with the DeLong method^9^. *P* values and AUCs significant after Bonferroni correction were indicated in bold (α = 0.05/4 = 0.01 for pairwise comparisons, α = 0.05/2 = 0.03 for AUCs). aMCI, amnestic mild cognitive impairment; AUC, area under curve; Aβ, β-amyloid; ROC, receiver operating characteristic; CN, cognitively normal; SIMOA, single molecule array; t-tau, total tau.

**Supplementary Figure 3.** **Correlations of ELISA and SIMOA plasma amyloid and tau levels with Centiloids and CSF Aβ_1-42_/t-tau**

**
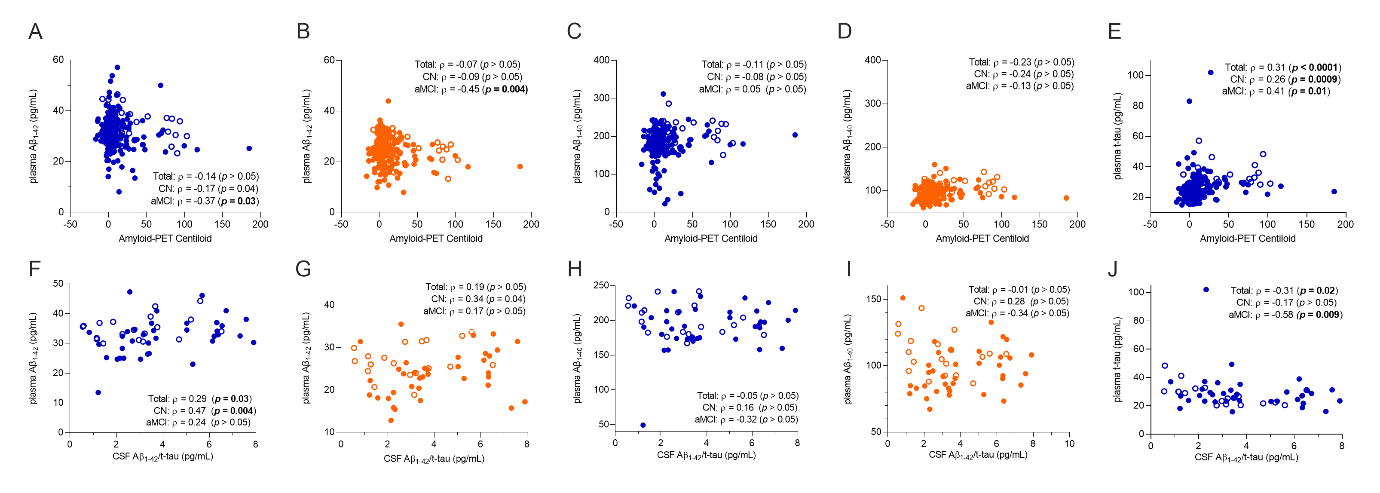
**

Plasma amyloid and tau levels were plotted against amyloid-PET Centiloid values (A-E) and CSF Aβ_1-42_/t-tau ratios (F-J). Spearman rank correlations were calculated in the total study population, as well as in the CN (n = 161 for PET, n = 37 for CSF) and aMCI subgroups (n = 38 for PET, n = 19 for CSF). Filled circles represent measurements in CN controls and open circles indicate measurements in aMCI patients of respectively Aβ_1-42_ (A,B,F,G), Aβ_1-40_ (C,D,H,I) and t-tau (E-J). Colours represent the employed immunoassay platform: blue for ELISA (A,C,E,F,H,J) and orange for SIMOA (B,D,G,I). *P* values are indicated in bold when correlations remained significant after correction for multiple comparisons (Bonferroni correction for the correlation of nine biomarkers with respectively PET and CSF: significance level α = 0.05/2 = 0.03). aMCI, amnestic mild cognitive impairment; Aβ, β-amyloid; t-tau, total tau; CN, cognitively normal; SIMOA, single molecule array

**REFERENCES**

1. The Global Alzheimer’s Association Interactive Network - The Centiloid Project. Available from: http://www.gaain.org/centiloid-project

2. Klunk WE, Koeppe RA, Price JC, Benzinger TL, Devous MD, Jagust WJ, et al. The Centiloid Project: standardizing quantitative amyloid plaque estimation by PET. Alzheimers Dement. Elsevier Inc; 2015;11:1-15.e1-4.

3. Battle MR, Pillay LC, Lowe VJ, Knopman D, Kemp B, Rowe CC, et al. Centiloid scaling for quantification of brain amyloid with [18F]flutemetamol using multiple processing methods. EJNMMI Res. 2018;8:107.

4. Rowe CC, Doré V, Jones G, Baxendale D, Mulligan RS, Bullich S, et al. 18F-Florbetaben PET beta-amyloid binding expressed in Centiloids. Eur J Nucl Med Mol Imaging. 2017;44:2053–9.
